# Sulforaphane Cannot Protect Human Fibroblasts From Repeated, Short and Sublethal Treatments with Hydrogen Peroxide

**DOI:** 10.3390/ijerph16040657

**Published:** 2019-02-23

**Authors:** Maria Chiara Lionetti, Federico Mutti, Erica Soldati, Maria Rita Fumagalli, Valentina Coccé, Graziano Colombo, Emanuela Astori, Alessandro Miani, Aldo Milzani, Isabella Dalle-Donne, Emilio Ciusani, Giulio Costantini, Caterina A. M. La Porta

**Affiliations:** 1 Center for Complexity and Biosystems, Department of Environmental Science and Policy, University of Milan, via Celoria 26, 20133 Milano, Italy; maria.lionetti@unimi.it (M.C.L.); federicomutti92@gmail.com (F.M.); ericasoldati92@gmail.it (E.S.); 2 Center for Complexity and Biosystems, Department of Physics, University of Milan, via Celoria 16, 20133 Milano, Italy; maria.fumagalli@unimi.it (M.R.F.); gr.costantini@gmail.com (G.C.); 3 Department of Biomedical, Surgical and Dental Sciences, University of Milan, Via Pascal 36, 20133 Milano, Italy; valentina.cocce@guest.unimi.it; 4 Department of Biosciences, University of Milan, via Celoria 26, 20133 Milano, Italy; graziano.colombo@unimi.it (G.C.); emanuela.astori@unimi.it (E.A.); aldo.milzani@unimi.it (A.M.); isabella.dalledonne@unimi.it (I.D.-D.); 5 Department of Environmental Science and Policy, University of Milan, via Celoria 10, 20133 Milano, Italy; alessandro.miani@unimi.it; 6 SIMA, Societá Italiana di Medicina Ambientale, via Monte Leone 2, 20149 Milano, Italy; 7 Fondazione IRCCS Istituto Neurologico C. Besta, Via Celoria 11, 20133 Milano, Italy; emilio.ciusani@istituto-besta.it

**Keywords:** oxidative stress, sulforaphane, fibrobalsts, p53

## Abstract

A delicate balance of reactive oxygen species (ROS) exists inside the cell: when the mechanisms that control the level of ROS fail, the cell is in an oxidative stress state, a condition that can accelerate aging processes. To contrast the pro-aging effect of ROS, the supplementation of antioxidants has been recently proposed. Sulforaphane (SFN) is an isothiocyanate isolated from Brassica plants that has been shown to modulate many critical factors inside the cells helping to counteract aging processes. In the present work, we exposed human dermal fibroblast to short, sublethal and repeated treatments with hydrogen peroxide for eight days, without or in combination with low concentration of SFN. Hydrogen peroxide treatments did not affect the oxidative status of the cells, without any significant change of the intracellular ROS levels or the number of mitochondria or thiols in total proteins. However, our regime promoted cell cycle progression and cell viability, increased the anti-apoptotic factor survivin and increased DNA damage, measured as number of foci positive for γ-H2AX. On the other hand, the treatment with SFN alone seemed to exert a protective effect, increasing the level of p53, which can block the expansion of possible DNA damaged cells. However, continued exposure to SFN at this concentration could not protect the cells from stress induced by hydrogen peroxide.

## 1. Introduction

Senescence is a complex process where the integrity and the structure of the nuclear scaffold changes [1]. One important factor contributing to cell senescence is oxidative stress [2].

Reactive oxygen species (ROS) are physiological by-products of mitochondria metabolism. Oxidative stress is due to an unbalanced oxidant/antioxidant status occurring in cells that could cause oxidative damage to DNA, lipids and proteins. ROS level regulates physiological functions, including signal transduction, gene expression, and proliferation, therefore they underlie physiological and pathological events [3]. For example, mitochondrial ROS may activate an adaptive response that promotes health to extend the lifespan through diseases prevention [4]. ROS overproduction, on the other hand, hampers nuclear and mitochondrial DNA repair at multiple steps, contributing to cell genomic instability [5]. Interestingly, ROS, including hydrogen peroxide, can inhibit cell growth and induce cell death and senescence in a context-dependent manner [6]. Accordingly, a recent paper reports that low levels of ROS can improve the defense mechanisms by inducing adaptive responses, which in turn contribute to stress resistance and longevity [7]. In contrast, high levels of ROS induce ineffective adaptive responses, contributing to aging onset and progression [7]. Many anti-aging strategies, from the scavenger of free radicals to the enhancing of antioxidant factors, are proposed to buffer the level of ROS.

The main goal of the present study was to investigate the impact of short and repeated sublethal treatments with hydrogen peroxide, commonly used to mimic oxidative stress [2], alone or in combination with sulforaphane (SFN) on human primary dermal fibroblasts (hSDF) focusing on critical biological functions of the cells. Recent evidence in fibroblasts shows that concentrations of 90–360 μM of hydrogen peroxide are sufficient to induce oxidative stress and premature cellular senescence in vitro recapitulating an aging process profile [8]. However, the possible protective effect of factors such as SFN on normal human cells such as human fibroblasts is not investigated in the literature yet. This kind of study appears crucial to plan possible advice in general for anti-aging purposes. We were not in fact strictly interested in skin aging but in general to develop a strategy to investigate the impact of specific factors on critical cellular functions linked to aging such as DNA damage.

SFN is a well tolerated natural compound obtained from cruciferous vegetables, which has been shown to have a cytoprotective effect through Nrf2-mediated induction of phase 2 detoxification and anti-oxidant enzymes, such as heme oxygenase-1 (HO-1), NAD[P] H:quinone oxidoreductase-1 (NQO1), superoxide dismutase (SOD), glutathione S-transferase (GST), and γ-glutamyl cysteine ligase (γ-GCL), which elevate cell defense against oxidative damage and promote the removal of potential carcinogens [9]. However, it is becoming clear that multiple mechanisms are activated in response to SFN, including the suppression of cytochrome P450 enzymes, the induction of apoptotic pathways, the suppression of cell cycle progression, and the inhibition of angiogenesis and anti-inflammatory activity [9,10,11]. Another important biological activity of SFN is the negative control of HDAC activity [9].

Altogether, our findings show that prolonged exposure to SFN alone increases p53 expression, suggesting that, in the absence of exogenous oxidative stress stimuli, it plays a protective role against DNA damage. The main significance and consequence of this findings is that everyday life can lead to a short and low increase of hydrogen peroxide repeated in time and SFN is not able to contrast these effects but exerts an anti-aging effect without oxidative stress changes. It is tempting to speculate that the combination of many factors rather than a single element can have a better protective effect. Further investigations will try to address these points.

## 2. Material and Methods

### 2.1. Cell Lines and Treatments

Human primary dermal fibroblasts (hSDF) (BS PRC 41, IZSLER, Brescia, Italy) were established from a skin biopsy obtained from a healthy adult donor during a surgical procedure [12] and cultured in EMEM (Euroclone) containing 1% L-Glutamine, 1% Penicillin/Streptomycin and 10% FBS (basal medium) at 37 ${}^{\circ }$C in 5% CO${}_{2}$ for no more than 10 passages [12]. The cells were treated with sublethal concentration of H${}_{2}$O${}_{2}$, for short (30 min) and repeated time [2].

Briefly, subconfluent cells were plated and exposed to 15 or 25 μM H${}_{2}$O${}_{2}$ (Fluka cod.95302) for 30 min at 37 ${}^{\circ }$C. This treatment was repeated every 48 h for four times (Figure S1). Untreated cells were plated and grown in basal medium for the entire duration of the experiment (eight days) (Figure S1). After every treatment with H${}_{2}$O${}_{2}$, the cells were washed twice with sterile PBS and maintained in basal medium until the next treatment. Subconfluent cells were treated with sulforaphane (SFN, cod.S4441, Sigma) at a final concentration of 1 μM for eight days after plating (see Figure S1). In combined experiments with H${}_{2}$O${}_{2}$ and SFN, cells were maintained in basal medium containing H${}_{2}$O${}_{2}$ for 30 min without SFN, and then replaced with fresh medium containing SFN (Figure S1).

### 2.2. Proliferation Assay

Sulforhodamine B (SRB) assay allows quantifying cellular protein content [13]. Briefly, the cells were fixed with 10% trichloroacetic acid (Sigma, cod.T6399) for 2 h at 4 ${}^{\circ }$C and 0.04% (wt/vol) SRB protein-bound dye (Sulforhodamine B Sigma, cod. S1402, dissolved in 10 mMTris base solution) was added to each well and incubated at RT for 1 h. After four washes with 1% (vol/vol) acetic acid, the samples were left to air-dry at room temperature. Then, 100 μL of 10 mM Tris base solution (pH 10.5) was added to each well and the plate was shaken on an orbital shaker for 10 min to solubilize the protein-bound dye. The absorbance at 510 nm was detected using an microplate reader (BioRad).

### 2.3. Cell Cycle Analysis

Subconfluent cells were harvested by trypsinization, pelleted and fixed in 70% cold ethanol and subsequently stained with propidium iodide (PI, cod. P4864, Sigma) for 30 min at 4 ${}^{\circ }$C [14]. PI fluorescence was analyzed using FACS Vantage SE Becton Dickinson flow cytometry. The percentages of cells in each phase of the cell cycle were calculated using FlowJO software.

### 2.4. p53 Level of Expression by Flow Cytometry

Subconfluent cells were fixed for 15 min in ice-cold methanol at −20 ${}^{\circ }$C, and incubated with primary antibody p53 linked to FITC at 4 ${}^{\circ }$C under dark condition (1:500, Abcam, ab156030) for 1 h and then immediately analyzed using FACS Vantage SE Becton Dickinson flow cytometry. Analysis were conducted using FlowJo software and the expression of p53 for each sample is reported as the ratio of the intensity of fluorescence with respect to unstained cells due to autofluorescence.

### 2.5. Quantification of Intracellular ROS by H2DCFDA

To detect ROS in cells, the cell-permeant 2’,7’-dichlorodihydrofluorescein diacetate (H2DCFDA) (Thermo Fisher, cod.D399) was used. It was converted into the highly fluorescent 2’,7’-dichlorofluorescein (DCF) by the cleavage of acetate groups due to intracellular esterases and oxidation. Briefly, acetylated dye was reconstituted in anhydrous dimethylsulfoxide (DMSO) at stock concentration of 100 μM just prior to use. Cells were incubated in 10 μM dye solution in pre-warmed PBS containing calcium and magnesium for 1 h at 37 ${}^{\circ }$C in 5% CO${}_{2}$, protected from light. Then, the loading buffer was removed and cells were returned to pre-warmed growth medium and incubated at the optimal temperature, for 1 h at 37 ${}^{\circ }$C in 5% CO${}_{2}$ to allow esterases to hydrolyze the acetate groups and render the dye responsive to oxidation. Fluorescence was determined using Ensight microplate fluorescence reader (Perkin Elmer) using Ex/Em: 492–495/517–527 nm. Results are reported as mean fluoresce values for each sample.

### 2.6. Quantification of Numbers of Mitochondria

To quantify the numbers of mitochondria per cell, MitoTracker probe was used. The MitoTracker probe passively diffuses across the plasma membrane and accumulates in active mitochondria. Lyophilized MitoTracker (Thermo Fisher, cod. M7512) was reconstituted in anhydrous dimethylsulfoxide (DMSO) to a final concentration of 1 mM, and then the cells were incubated in 250 nM MitoTracher probe solution in pre-warmed growth medium for 45 min at 37 ${}^{\circ }$C in 5% CO${}_{2}$ under dark condition. Fluorescence was detected using FACS Vantage SE Becton Dickinson flow cytometry and the data were analyzed by FlowJo software. Results are reported as the ratio of the intensity of fluorescence of each sample with respect to unstained cells due to autofluorescence.

### 2.7. Quantification of Thiols in Proteins

Total cellular proteins were obtained by cell lysis with ice-cold lysis buffer (50 mM Tris-HCl, pH 7.4, 150 mM NaCl, 1% TRITON X-100, 0.1% SDS, 0.5% sodium deoxycholate supplemented with protease inhibitors). The lysate was incubated on ice for 30 min and centrifuged at 10,000 rpm for 10 min at 4 ${}^{\circ }$C to remove cell debris. The concentration of protein was assessed using BCA protein assay. To detect thiols present in proteins, a biotin-maleimide assay was carried out. Briefly, 40 mM biotin-maleimide stock solution was prepared in DMSO and stored at −20 ${}^{\circ }$C. Then, 1 mg/mL of protein was incubated with 75 μM biotin-maleimide solution for 1 h at RT and mixed to Laemmli sample buffer (2% SDS, 20% glycerol, and 125 mM Tris-HCl, pH 6.8), boiled for 5 min at 90 ${}^{\circ }$C and immediately loaded on 12% SDS-PAGE gel [15]. The proteins were then electroblotted onto a low-fluorescence polyvinylidene difluoride (LF-PVDF) membrane. Biotin tag was revealed using streptavidin-HRP assay as following. LF-PVDF membrane was washed with PBST (10 mM Na-phosphate, pH 7.2, 0.9% (*w*/*v*) NaCl, 0.1% (*v*/*v*) Tween-20 (Sigma Aldrich, cod. P9416) [15] and blocked for 1 h in 5% (*w*/*v*) non-fat dry milk in PBST. After washing three times with PBST for 5 min, biotin tag was probed by 2-h incubation with 5% non-fat dry milk/PBST containing streptavidin-HRP (1:5000 dilution, BioRad). Biotinylated proteins were visualized by ECL detection (cod.1705061, Biorad) using Chemidoc Touch Imaging System (Biorad). ECL signals were normalized with respect to PVDF stain free [16].

### 2.8. Western Blot

Subconfluent cells were lysed in Laemmli sample buffer (2% SDS, 20% glycerol, and 125 mM Tris-HCl, pH 6.8), briefly centrifuged and the protein concentration of the supernatant was measured by BCA Protein Assay Kit (cod.23225, Thermo Scientific). Then 15 μg protein were loaded on 12% SDS-PAGE gel and transferred on PVDF using the TranBlot Turbo Transfer System Bio-Rad (cod.1704150, BioRad). After incubation of the PVDF sheet with 5% fat dry milk (cod.70166, Sigma) in PBS Tween 0.1% for 1 h at room temperature, the membrane was incubated overnight at 4 ${}^{\circ }$C with primary antibody, anti-Nrf2 (1:2000, ADI-KAP-TF125, ENZO). As housekeeping gene, anti-βactin (1:10,000, ab11003, Abcam) was used. The sheet was then incubated with secondary antibodies, anti-rabbit HRP (Bio-Rad 1: 3000) or anti-Mouse HRP (Bio-Rad 1: 3000) for 1 h. ECL Blotting reagents (GE Healthcare, cod. RPN2109) were used at room temperature to detect chemioluminescence. The signal was acquired using Chemidoc Touch (cod. 1708370, Bio-Rad). Densitometric analysis was carried out with ImageJ.

### 2.9. Immunofluorescence

Subconfluent cells plated on coverslips were fixed with 3.7% paraformaldehyde (BDH 29447) or in 100% cold methanol. For paraformaldehyde fixed cells, the cells were permeabilized with 0.1 %TritonX-100 in PBS for 15 min at RT and then incubated with blocking solution (1%BSA/10% goat-serum/0.3 M glycine/0.1% Tween in PBS) for 1 h at RT. The cells were incubated overnight at 4 ${}^{\circ }$C with the primary antibody as following: γ-H2AX (1:700, Abcam, ab2893-Phospho139, Rabbit) or anti-survivin (1:250, NB500-201, Novus Biological, Rabbit). The samples were incubated with secondary antibodies FITC anti-Rabbit (1:250, ab150077, AbCam) for 1 h at RT and then mounted with Pro-long anti-fade reagent (P7481, Life Technologies) with DAPI to stain the nuclei. The images were acquired with a Leica TCS NT confocal microscope.

### 2.10. γ-H2AX Spots Counting and Nuclear Survivin

γ-H2AX spots inside the nuclei were counted using spot detector tool of ICY Software as described in our previous paper [17]. Briefly, we created a ROI for each nucleus and we computed the number of the marker spots inside its enabling the Scale n.3 with a sensitivity equal to 15. We also extracted the number of nuclei from the images to calculate the ratio of the number of foci per nucleus. The level of expression of survivin fluorescence inside the nucleus was evaluated using a custom pipeline in ICY software. Briefly, we created a ROI for each nucleus and evaluated the mean intensity of Survivin signal over all the nuclear surface. DAPI channel intensity was considered to verify the absence possible bias due to differences between the nuclei and images.

### 2.11. Statistical Analysis

Statistical significance analyses were performed using the Kolmogorov–Smirnov test and unpaired t-test.

## 3. Results

### 3.1. Effect of Short and Repeated Sublethal Treatment with Hydrogen Peroxide without or in Combination with SFN on the Oxidative Status of hSDF

**Figure 1:**
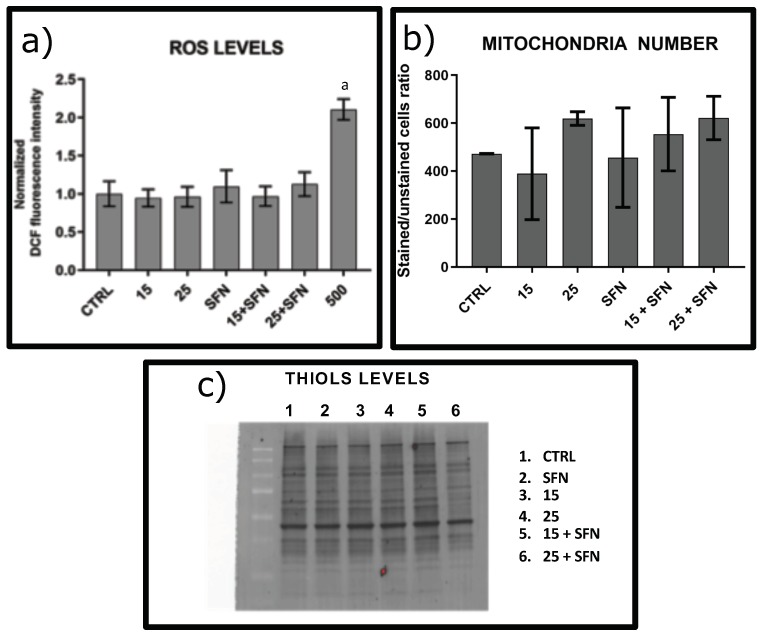
Evaluation of oxidative status after treatment with sublethal concentration and repeated exposure with hydrogen peroxide without or with 1 μM SFN. (**a**) ROS level Subconfluent cells treated as described in Figure S1 and Material and Methods Section with 15 or 25 μM hydrogen peroxide (15 or 25) without or with 1 μM SFN (15+SFN, or 25+SFN, respectively) up to eight days and incubated in 10 μM H2DCFDA (Thermo Fisher, cod.D399) in pre-warmed PBS for 1 h at 37 ${}^{\circ }$C in 5% CO${}_{2}$. Cells were also treated with 500 μM hydrogen peroxide for 1 h and then the fluorescence was immediately quantified (positive control). Fluorescence was determined using Ensight microplate fluorescence reader (Perkin Elmer) using Ex/Em: 492–495/517–527 nm. Results are reported as mean fluoresce values for each sample. Each bar represents the mean and the corresponding error bars of 16 independent measures for all the treatments and untreated cells with the exception of 500 μM hydrogen peroxide where we carried out four independent measurements. a: *p* < 0.0001 versus untreated cells. (**b**) Numbers of Mitochondria: Cells treated as described in (**a**) were quantified using MitoTracker probe, which passively diffuses across the plasma membrane and accumulates in active mitochondria. Subconfluent cells were incubated with 250 nM MitoTracker (Thermo Fisher, cod. M7512) for 45 min at 37 ${}^{\circ }$C in 5% CO${}_{2}$. Fluorescence was detected using FACS Vantage SE Becton Dickinson flow cytometry and the data were analyzed by FlowJo. Results are reported as the ratio between the intensity of fluorescence of each sample with respect to unstained cells due to autofluorescence. The bars are the mean with the statistic errors of three independent experiments. (**c**) Levels of thiols into total protein. Total cellular proteins were obtained by cell homogenization with ice-cold lysis buffer. The lysate was incubated on ice for 30 min and centrifuged at 10,000 rpm for 10 min at 4 ${}^{\circ }$C to remove cell debris. The concentration of protein was assessed using BCA protein assay. To detect thiols present into proteins a biotin-maleimide assay was carried out. First, 1 mg/mL of protein was incubated with 75 μM biotin-maleimide solution for 1 h at RT and then mixed to Laemmli sample buffer, boiled for 5 min at 90 ${}^{\circ }$C and immediately loaded on 12% SDSPAGE gel. The proteins were then electroblotted onto a low-fluorescence polyvinylidene difluoride (LF-PVDF) membrane. Biotin tag was revealed using streptavidin-HRP assay. Biotinylated proteins were visualized by ECL detection (cod.1705061, Biorad) using Chemidoc Touch Imaging System (Biorad). ECL signals were normalized with respect to PVDF stain free. This gel is representative of four independent experiments carried out.

H2DCFDA is a chemically reduced form of fluorescein used as an indicator for intracellular ROS levels. The short oxidizing treatment (30 min) repeated every 48 h for eight days with sublethal concentrations (15 μM or 25 μM) of hydrogen peroxide (see Figure S1) according to Caldini et al. [2] alone or in combination with 1 μM SFN does not affect the levels of ROS measured using H2CDFDA assay or the numbers of mitochondria in the cells quantified by flow cytometry (Figure 1). These data suggest that, during the 48 h of recovering, hSDF cells implemented response and adaptive mechanisms to protect against permanent injuries. Since it is known that Nfr2 is a transcription factor whose activation is induced by SNF [18,19], we performed Western blot of Nrf2 on untreated cells with respect to cells treated with SFN for 8days. As shown in Figure S2, we found a significative increase in the level of Nrf2 in treated cells (*p* < 0.01). Furthermore, we also checked the ability of these cells to be affected by high levels of ROS using H2DCFDA reduction as an indicator of the intracellular ROS level, as shown in Figure 1. The treatment with 500 μM hydrogen peroxide for 1 h doubled the level of ROS (*p* < 0.0001), confirming that the cells responded to hydrogen peroxide induction.

It is known that oxidative stress leads to the formation of unwanted disulfide bonds in the cytoplasm, eventually leading to impaired protein function. To face this, the cells have several mechanisms to increase the intracellular levels of thiols [20]. Notably, intracellular increase of thiol levels are strongly associated with an increased tolerance to an oxidant stress [20] since they act as extraordinarily efficient antioxidants protecting the cells against consequences of damage induced by ROS [21]. Differently, an age-dependent reduction in the amount of (free) thiols occurs in plasma proteins in healthy humans. This indicates that the efficiency of the reduced protein thiol pool as an antioxidant defense system decreases with age. The drop in the plasma level of protein thiol suggests depletion and/or impairment of the antioxidant capacity of plasma [22]. Indeed, the protein thiolation index, i.e., the molar ratio between the sum of all low molecular mass thiols bound to plasma proteins (forming, as a whole, S-thiolated proteins) and protein free thiols, is a suitable biomarker of oxidative stress [23]. Protein thiolation index shows a near linear age-dependent increase during aging in humans and is a useful indicator of thiol-specific oxidative stress in patients with end stage renal disease on maintenance hemodialysis [24]. Under our experimental conditions, the levels of reduced thiols in total proteins measured by biotin maleimide assay did not show any significant change (Figure 1).

### 3.2. SFN and Oxidative Stress Decrease Cell Vitality and Regulate Apoptosis

**Figure 2:**
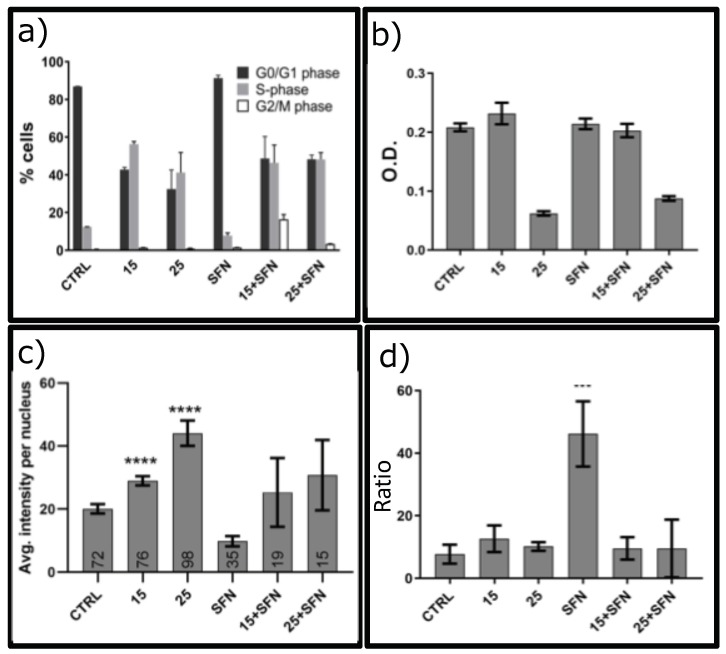
Effect on cell vitality, PI staining and Survivin expression of sublethal concentration and repeated exposure to hydrogen peroxide without or with 1 μM SFN. The subconfluent treated cells as described in Figure S1 and Material and Methods Section with 15 or 25 μM hydrogen peroxide (15 or 25) without or with 1 μM SFN (15+SFN, or 25+SFN, respectively) up to eight days. (**a**) Propidium Iodide (PI) staining: Subconfluent cells were fixed in 70% cold ethanol and stained with PI (cod. P4864, Sigma) for 30 min at 4 ${}^{\circ }$C. PI fluorescence was analyzed using FACS Vantage SE Becton Dickinson flow cytometry. The percentages of cells in each phase of the cell cycle were calculated using FlowJo software.The bars show the mean and the statistic errors of two independent experiments. (**b**) Proliferation assay: Sulforhodamine B (SRB) assay allowed quantifying cellular protein content. Briefly, cells were fixed with 10% Trichloroacetic acid (Sigma, cod.T6399) for 2 h at 4 ${}^{\circ }$C. 0.04% (wt/vol) SRB protein-bound dye was added to each well and incubated at RT for 1 h. Then, 100 μL of 10 mM Tris base solution (pH 10.5) was added to each well and the plate placed on an orbital shaker for 10 min to solubilize the protein-bound dye. Absorbance was detected using a Ensight microplate reader (Perkin Elmer) at 510 nm. The bars are the mean and statistic errors of at least 18 independent sample. **** *p* < 0.0001 versus untreated cells. (**c**) Immunofluorescence of Survivin. Subconfuent cells plated on coverslips were fixed with 3.7% paraformaldehyde, permeabilized with 0.1%TRITOX-100 in PBS for 15 min at RT, and incubated overnight at 4 ${}^{\circ }$C with anti-Survivin (1:250, NB500-201, Novus Biological). The samples were incubated with the secondary antibody FITC anti-Rabbit (1:250, ab150077, AbCam) and then mounted with Pro-long anti-fade reagent (P7481, Life Technologies) with DAPI to stain the nuclei. The images were acquired with a Leica TCS NT confocal microscope. Here is shown the quantification of the nuclear fluorescence (see Materials and Methods). In the bars are reported the number of cells analyzed for each conditions of at least two independent experiments. (**d**) p53 level of expression by flow cytometry: Subconfluent cells were fixed for 15 min in ice cold methanol at −20 ${}^{\circ }$C, and then incubated with primary antibody anti-p53 FITC-conjugated at 4 ${}^{\circ }$C (1:500, Abcam, ab156030 Mouse) for 1 h and then immediately analyzed using FACS Vantage SE Becton Dickinson flow cytometry. Analysis were conducted using FlowJo software and the expression of p53 for each sample is reported as the ratio between the intensity of fluorescence with respect to unstained cells due to autofluorescence. Two independent experiments were carried out, each in triplicate.

The short treatment (30 min) with H${}_{2}$O${}_{2}$ repeated every 48 h for eight days with sublethal concentrations (15 μM or 25 μM) (Figure S1) impacted cell cycle profile of hSDF cells, as shown in Figure 2a. In fact, we observed a shift of distribution towards S-phase in all experimental conditions and a slight increase in the number of cells into G2-M phase when treated with hydrogen peroxide combined with SFN (Figure 2a). On the other hand, the treatment with alone SFN did no result in restoring the typical cell cycle pattern distribution of these cells and in combination with hydrogen peroxide does not protect from the effect due to oxidative stress (Figure 2a). Since the cells were not synchronized, it is tempting to speculate that the number of cells that are in a certain cell cycle phase is proportional to the time that cells spend in that phase of the cell cycle. We also detected the viability of the cells with the SRB assay. As shown in Figure 2b, hSDFs viability decreased significantly with 25 μM of H${}_{2}$O${}_{2}$ alone. The cytotoxic effect of 25 μM of H${}_{2}$O${}_{2}$ was not prevented by the presence of SFN (Figure 2b).

To investigate whether this regime changed the apoptotic pathway, we analyzed the expression of a well known anti-apoptotic factor, survivin. Figure 2c and Figure S3 show an increased level of expression of survivin in hSDF cells treated with both concentrations of hydrogen peroxide in the absence and presence of SFN. Moreover, the treatment with SFN alone did not affect survivin expression (Figure 2c and Figure S3). We also checked the presence in the medium of apoptotic cells every 48 h when we changed the medium of the cells with fresh one after hydrogen peroxide treatment. We found always less than 4% of apoptosis.

Finally, we checked p53 expression, a well known protein that controls the genome by orchestrating a variety of DNA-damage responses to restore genome stability and that plays a critical role in triggering apoptotic pathways in damaged cells [25]. Interestingly, the treatment with 1 μM SFN alone increased significantly the level of expression of total p53 (Figure 2d). This effect disappeared when the cells were exposed to both SFN and hydrogen peroxide (Figure 2d).

### 3.3. Effect of SFN Alone or with Hydrogen Peroxide on DNA Damage

**Figure 3:**
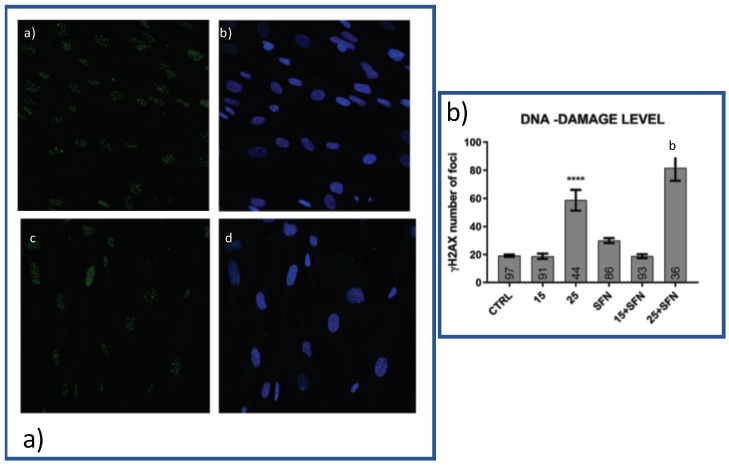
Effects of SFN alone or in combination with hydrogen peroxide on DNA-damage Subconfluent cells were treated as described in Figure S1 and Material and Methods Section. (**a**) An example of immunofluorescence obtained with cells untreated (CTRL) and treated with 25 μM hydrogen peroxide in presence or absence of SFN. Scale bar is 10 μM. Untreated cells (**a**,**b**); and treated ones (**c**,**d**) stained for γH2AX (1:700, Abcam, ab2893-Phospho139) (**a**,**c**) or both for γH2AX and DAPI (**b**,**d**). Cells were fixed with 3.7% paraformaldehyde, permeabilized with 0.1%TRITOX-100 in PBS for 15 min at RT and incubated overnight at 4 ${}^{\circ }$C with the γH2AX (1:700, Abcam, ab2893-Phospho139). The samples were then incubated with FITC anti-Rabbit (1:250, ab150077, AbCam) for 1 h at RT and mounted with Pro-long anti-fade reagent (P7481, Life Technologies) with DAPI to stain the nuclei. The images were acquired with a Leica TCS NT confocal microscope. Panel b shows γH2AX spots inside the nuclei counted using spot detector tool of ICY Software as described in the Materials and Method section. All the resulting values are normalized with the total number of pixels of their image, to make possible the comparison of all the nuclei, one with each other. In the bars are reported the number of cells analyzed for each conditions. **** *p* < 0.0001 versus untreated cells; b: *p* < 0.0001 versus SFN treated cells.

Histone γ-H2AX is the most sensitive marker of double-stranded DNA breaks (DSB) and telomere shortening [26]. Herein, we quantified the number of γ-H2AX foci in hSDF cells after eight days of hydrogen peroxide treatment with or without 1 μM SFN. As shown in Figure 3, there was a significant increase in the number of γ-H2AX positive foci, increasing the concentration of hydrogen peroxide. In SFN treated hSDF cells, there was no significant change in comparison to the untreated cells (Figure 3).

## 4. Discussion

Sulforaphane (SFN) is mainly present in Cruciferae such as broccoli sprouts and cabbages. It is a very well tolerated factor, showing antioxidant properties and inhibiting histone deacetylase enzymes (HDAC) [9].

SFN seems to have a double-faced effect: on the one hand, it helps the clearance of progerin in accelerating aging [27], and, on the other hand, it acts as anti-tumorigenic factor targeting cancer stem cells (CSC) [10,28]. Furthermore, high levels of SFN (higher than 5 μM) were shown to induce apoptosis in cancer cells increasing ROS [11]. However, very little is known about the effects of SFN on healthy human cells. In a recent study, the effect of SFN on human mesenchymal stem cells (MSCs) at different concentrations has been investigated [29], resulting in contrasting effects. In fact, while low (1 μM) doses of SFN for three days enhanced the cellular proliferation and protected the cells against apoptosis and senescence, higher (5 μM) concentrations had a cytotoxic effect, leading to cell cycle arrest, programmed cell death and senescence [29]. Some ROS, mainly hydrogen peroxide, at sublethal concentrations act as second messenger in signaling cascades and are involved in cell proliferation and differentiation [30,31]. It has been recently reported that moderate increases in ROS levels trigger signaling pathways involved in cell proliferation, whereas an excessive ROS increase causes oxidative stress, which in turn induces cell death and/or senescence [32].

The main goal of this study was to investigate the combined effect of sublethal concentrations and long-term exposure to SFN and $H_{2}O_{2}$ on human primary normal dermal fibroblasts (hSDF) on critical cell functions and the possible protective role of SFN against negative effects of oxidative stress. Regarding to hydrogen peroxide, we used a physiological concentration [33]. Our experimental approach led to faithfully mimicking physiologic stress conditions. In fact, in the majority of the studies present in the literature, the experimental induction of oxidative stress is achieved by short exposure of the cells to high concentration of exogenous ROS, or by long term and continuous exposure to moderate concentration of exogenous ROS. Both models are unlikely to reproduce physiologic conditions, where stimuli are discontinuous and ROS exposure limited. Indeed, excluding particular pathological conditions, it is very rare to find constantly increased level of ROS in healthy people but rather occasional and short ROS levels increases, albeit for a long time [34]. The sublethal exposure to hydrogen peroxide repeated for 30 min every 48 h up to eight days does not significantly change the oxidative status of the cell measured as levels of ROS, number of mitochondria and levels of thiols in total proteins. This suggests that, using our protocol, the cells are able to activate compensatory mechanisms and recover the physiological oxidative status. However, hydrogen peroxide, both alone or in combination with SFN, modifies the complex and delicate physiology of the cells since it promotes the cell cycle, contrasts apoptosis increasing Survivin expression albeit without changing p53 levels. Moreover, hydrogen peroxide exposure results in a higher number of γ-H2AX positive foci, which quantified DNA damage.

Two interesting results are related to SFN. Firstly, SFN induces alone an increase of p53 but does not induce any DNA damages. Consistently, the presence of SFN upregulates and stabilizes p53 oscillatory physiologic behavior probably due to its indirect effect on NRF2 and HIPK2 [35,36,37]. In fact, SNF decreases the ubiquitinization of Nrf2 [38], which leads to Nrf2 to translocate into the nucleus where it can accumulate and activate its target genes [37]. In particular, HIPK2 is transcriptionally regulated by Nrf2 [35] and its overexpression downregulates WIP1 participating to a negative feedback loop with p53 [39,40]. A direct consequence is an increase of p53 level and a stabilization of its oscillatory dynamics [36,40,41]. Moreover, in our experimental conditions, the presence of hydrogen peroxide stimulus prevent the SFN-induced increase of p53 possibly due to the activation of different response pathways p53 independent.

The second interesting result is that SFN cannot counteract the effect of hydrogen peroxide in hSDFs, confirming SFN negligible scavenging capacity [42] but also suggesting the presence of a common mechanism of action that results in cell type-specific response of either cell death and survival. In non-cancer cells, which have an inherent ROS level (IRL) lower than for cancer cells, SFN exposure causes just an adaptive antioxidant response, whereas, in cancer cells, which have an IRL closer to the ROS death threshold, leads to growth inhibition and death [42]. In conclusion, our findings show that SFN is not able to protect against low concentration and repeated exposure to hydrogen peroxide in human fibroblasts cells resembling a physiological condition of everyday life. Further studies should investigate the possible effect of synergic factors to protect these kinds of cellular damages. In fact, to untangle the complex network inside the cells, it is necessary to investigate the co-exposure to multiple factors in a model such as ours that resembles a physiological condition.

## Supplementary Materials

The following are available at https://www.mdpi.com/1660-4601/16/4/657/s1. Figure S1: Schematic time line of the treatments with hydrogen peroxide alone or in combination with SFN, Figure S2: Level of expression of Nrf2 in SFN treated cells, Figure S3: Immunoflorescence of survivin.
