# Supplementary material for: Sulforaphane Cannot Protect Human Fibroblasts From Repeated, Short and Sublethal Treatments with Hydrogen Peroxide
Source: Int J Environ Res Public Health. 2019 Feb 23;16(4):657. doi: 10.3390/ijerph16040657 (PMC6406632; doi:10.3390/ijerph16040657)
Supplement: Supplementary file 1 [file ijerph-16-00657-s001.pdf]

International Journal of
Environmental Research and Public Health

MDPI

Article

Sulforaphane Can Not Protect Human Fibroblasts From Repeated, Short and Sublethal Treatments with Hydrogen Peroxide

Maria Chiara Lionetti [1], Federico Mutti [1], Erica Soldati [1], Maria Rita Fumagalli [2], Valentina Coccé [3], Graziano Colombo [4], Emanuela Astori [4], Alessandro Miani [5,6], Aldo Milzani [4], Isabella Dalle-Donne [4], Emilio Ciusani [7], Giulio Costantini [2] and Caterina A. M. La Porta [1,]*

1 Center for Complexity and Biosystems, Department of Environmental Science and Policy, University of Milan, via Celoria 26, 20133 Milano, Italy; maria.lionetti@unimi.it (M.C.L.); federicomutti92@gmail.com (F.M.); erica.soldati@studenti.unimi.it (E.S.)
2 Center for Complexity and Biosystems, Department of Physics, University of Milan, via Celoria 16, 20133 Milano, Italy; maria.fumagalli@unimi.it (M.R.F.); gr.costantini@gmail.com (G.C.)
3 Department of Biomedical, Surgical and Dental Sciences, University of Milan, Via Pascal 36, 20133 Milano, Italy; valentina.cocce@guest.unimi.it
4 Department of Biosciences, University of Milan, via Celoria 26, 20133 Milano, Italy; graziano.colombo@unimi.it (G.C.); emanuela.astori@unimi.it (E.A.); aldo.milzani@unimi.it (A.M.); isabella.dalledonne@unimi.it (I.D.-D.)
5 Department of Environmental Science and Policy, University of Milan, via Celoria 10, 20133 Milano, Italy; alessandro.miani@unimi.it
6 SIMA, Societá Italiana di Medicina Ambientale, via Monte Leone 2, 20149, Milano, Italy
7 Istituto Besta, Via Celoria 11, 20133 Milano, Italy; emilio.ciusani@istituto-besta.it
* Correspondence: caterina.laporta@unimi.it

Received: date; Accepted: date; Published: date

check for updates

Abstract: A delicate balance of reactive oxygen species (ROS) exists inside the cell: when the mechanisms that control the level of ROS fail, the cell is in an oxidative stress state, a condition that can help aging processes. To contrast the pro-aging effect of ROS, the supplementation of antioxidants has been recently proposed. Sulforaphane (SFN), is an isothiocyanate isolated from Brassica plants that has been shown to modulate many critical factors inside the cells helping to counteract aging process. In the present work, we exposed human dermal fibroblast to short, sublethal and repeated treatments with hydrogen peroxide for eight days, without or in combination with low concentration of SFN. Hydrogen peroxide treatments do not affect the oxidative status of the cells, without any significant change of the intracellular ROS levels or the number of mitochondria or thiols in total proteins. However, our regime promotes cell cycle progression and cell viability, increases the anti-apoptotic factor Survivin, decreases Lamin B1 and increases DNA damage, measured as number of foci positive for γ-H2AX. On the other hand, the treatment with SFN alone seems to exert a protective effect increasing the level of p53 than can block the expansion of possible DNA damaged cells. However, continued exposure to SFN is not able at this concentration to protect the cells from stress induced by hydrogen peroxide.

Keywords: oxidative stress; sulforaphane; fibrobalsts; lamin

Supplementary Materials

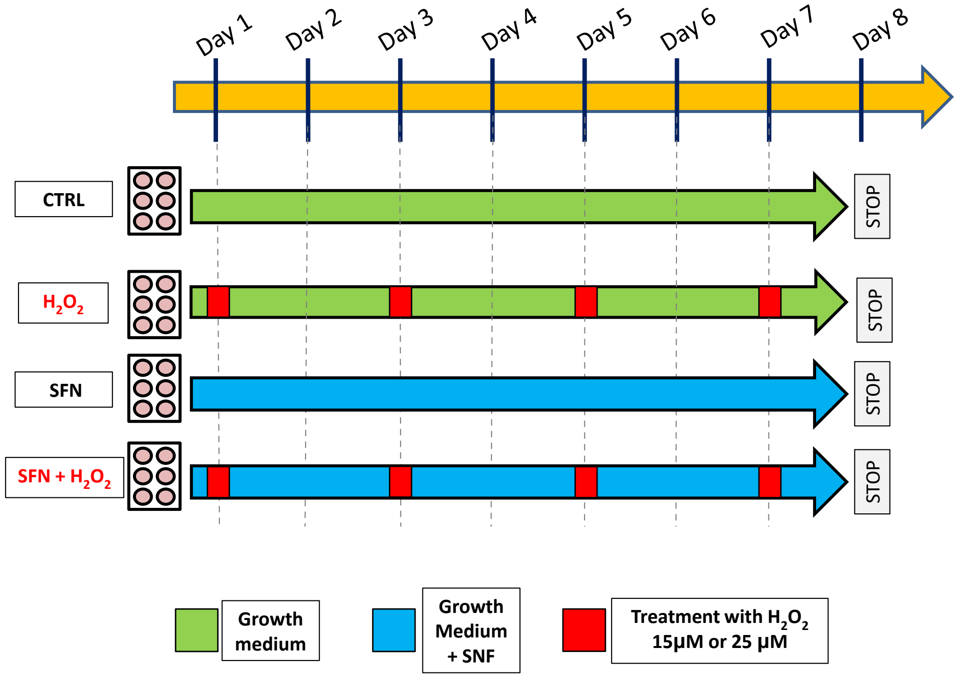

Figure S1. Schematic time line of the treatments with hydrogen peroxide alone or in combination with SFN.

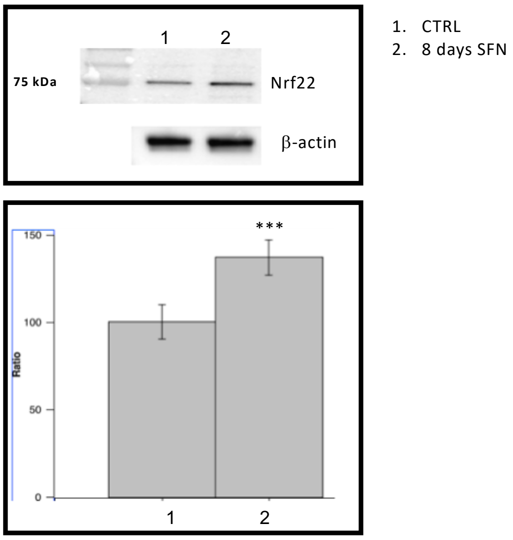

Figure S2. Level of expression of Nrf2 in SFN treated cells. Protein obtained from subconfluent cells (15 μg) treated for eight days with 1 μM SFN were loaded on 12% SDS-PAGE gel and transferred on PVDF using the TranBlot Turbo Transfer System Bio-Rad. The membrane was incubated overnight at 4 °C with anti-Nrf2 (1:2000, ENZO cod. ADI-KAP-TF125). Anti-*beta*actin antibody (1:10,000, ab11003, Abcam) was used as housekeeping. Signals were quantified by densitometric analysis using ImageJ. *** $p < 0.01$ versus untreated cells.

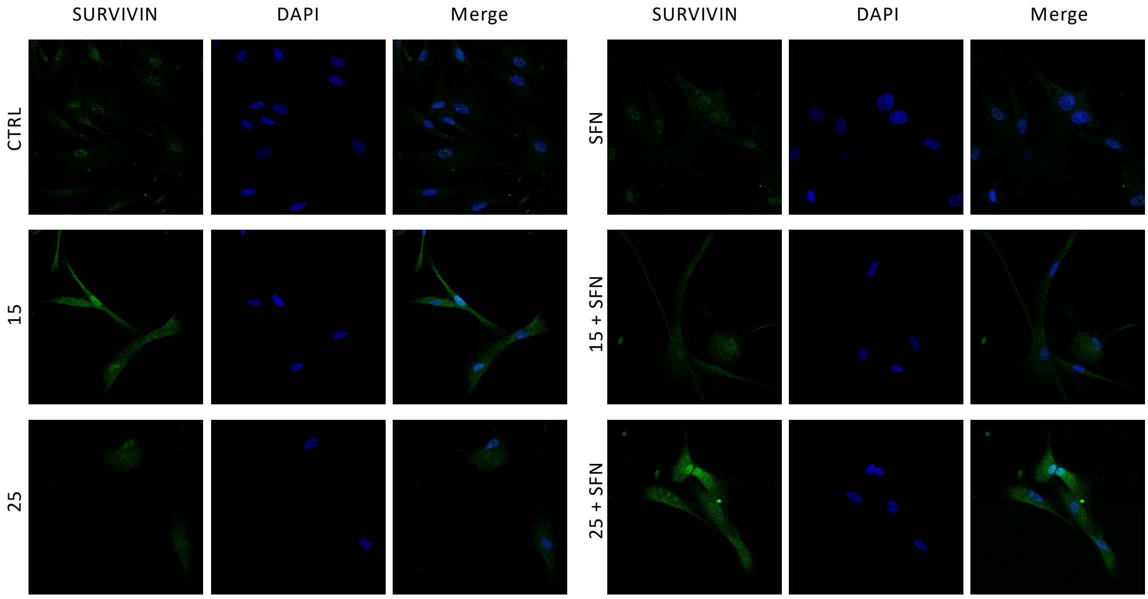

Figure S3. Immunoflorescence of survivin. Subconfuent cells (untreated or treated with hydrogen peroxide or SFN alone or in combination) were plated on coverslips, fixed with 3.7% paraformaldehyde and incubated overnight at 4 °C with anti-Survivin (1:250, NB500-201, Novus Biological). The images were acquired with a Leica TCS NT confocal microscope. Here is shown the quantification of the nuclear fluorescence (see Materials and Methods). The bars show the mean and statistic errors of at least two independent experiments.

© 2019 by the authors. Licensee MDPI, Basel, Switzerland. This article is an open access article distributed under the terms and conditions of the Creative Commons Attribution (CC BY) license (http://creativecommons.org/licenses/by/4.0/).
